# Unobtrusive Human Activity Recognition Using Multivariate Indoor Air Quality Sensing and Hierarchical Event Detection

**DOI:** 10.3390/s26092857

**Published:** 2026-05-02

**Authors:** Grigoriοs Protopsaltis, Christos Mountzouris, Gerasimos Theodorou, John Gialelis

**Affiliations:** Department of Electrical and Computer Engineering, University of Patras, 26504 Patras, Greece; g.protopsaltis@ac.upatras.gr (G.P.); mountzou@ece.upatras.gr (C.M.); gtheodorou@teemail.gr (G.T.)

**Keywords:** indoor air quality, activity recognition, hierarchical classification, environmental monitoring

## Abstract

**Highlights:**

**What are the main findings?**

- Indoor human activities can be inferred from indoor air quality measurements.
- A gated hierarchical model separates activity detection from classification, achieving high activity detention recall and conditional classification accuracy.

**What are the implications of the main findings?**

- Indoor pollutant dynamics contain sufficient information to enable unobtrusive activity recognition based on environmental sensing only.
- The proposed framework supports intelligent control applications, such as adaptive ventilation and health-aware indoor environmental control.

**Abstract:**

Recent studies have shown that common household activities produce characteristic patterns in indoor air pollutants, enabling activity inference using environmental measurements alone. However, pollutant-based approaches are usually formulated as flat multi-class classification problems, even though indoor environments are dominated by long baseline periods with no emission-generating activity, leading to false alarms and unstable predictions. This work proposes a gated hierarchical inference framework for recognizing activities from indoor air quality data. A first-stage gate detects whether a time window contains activity-induced pollutant dynamics, while a second-stage classifier conditionally identifies the specific activity only when activity relevance is detected. Multivariate time-series measurements of particulate matter, volatile organic compounds, nitrogen oxides, carbon dioxide, temperature and relative humidity were collected using a portable monitoring system during controlled household cooking and cleaning experiments. Temporal windows were processed using recurrent neural network models in both stages. By separating activity detection from activity identification, the proposed method aligns inference with the physical generation of indoor pollutant signals and improves robustness in baseline-dominated monitoring scenarios while maintaining reliable discrimination among activities. The framework supports unobtrusive activity recognition and enables applications in exposure-aware monitoring and intelligent indoor environmental management.

## 1. Introduction

Human activity recognition (HAR) in indoor environments has traditionally relied on sensing modalities such as cameras, microphones, wearable devices and other contact-based or proximity-based systems. Continuous audio/visual sensing may be perceived as intrusive, can capture identifiable information and therefore is frequently resisted in domestic environments. Beyond privacy, real-world installations may face operational barriers, including device placement constraints, calibration needs, network dependence and ongoing maintenance, which can reduce long-term robustness and user adoption [1].

A complementary and less intrusive alternative is to infer indoor activities from changes in the parameters that affect the indoor air quality (IAQ). Many common household actions directly affect pollutant concentrations and produce measurable temporal signatures in the indoor environment. Cooking can generate strong particulate matter (PM), volatile organic compound (VOC) and combustion-related pollutant spikes, while cleaning activities may elevate VOCs and in some cases coarse and fine particles through product use or particle resuspension previously deposited on various surfaces. Because these activities alter the chemical and physical composition of the air in different ways, it is theorized that multivariate IAQ measurements can provide proxies for activity inference, all while preserving the privacy of the occupant [2,3].

This sensing paradigm has recently gained explicit attention, and approaches combining indoor environmental monitoring and HAR have emerged. Gerina et al. demonstrated that cooking-related behavior can be recognized using air quality sensor datastreams, showing that pollutant measurements can support unobtrusive and privacy-preserving detection of food-preparation events in real-life home environments [4]. More recently, Miranda et al. proposed an automatic detection framework for indoor air-pollution-related activities using metal-oxide gas sensors and time-windowed analysis, further confirming that environmental sensing alone can identify activity-linked pollution events without relying on intrusive modalities [5]. Building on this direction, Mentou et al. investigated the relationship between indoor pollutant dynamics and household activities, formulating activity recognition directly on temporal patterns of pollutants measured near the human breathing zone to reinforce the feasibility of this approach for everyday indoor scenarios as well as quantify the risk of exposure to certain levels of the pollutants [6]. More broadly, recent machine-learning studies in exposure science have shown how air-quality observations can be transformed into spatiotemporal exposure and health-risk information, further supporting the relevance of data-driven environmental inference as a broader context for pollutant-based activity recognition [7].

Beyond recognizing activities, pollution events are increasingly treated as actionable triggers for ventilation and air-cleaning control. Sensor-driven control strategies during cooking have been shown to reduce indoor PM_2.5_ exposure when interventions such as stove hoods and portable air cleaners are activated based on real-time measurements [8]. In parallel, indoor air chemistry studies demonstrate that cooking and cleaning emit complex VOC mixtures and can drive secondary chemistry whose evolution depends on ventilation, oxidant levels and gas-surface interactions [9,10,11]. These findings motivate exposure-oriented sensing close to the breathing zone and complement evidence that wearable air pollution sensors can capture spatiotemporal variability relevant to personal exposure assessment [12]. Moreover, smart-building research indicates that non-intrusive environmental variables (e.g., CO_2_, differential pressure, ventilation state) can be leveraged to infer occupancy-related states without intrusive sensing [13], while recent reviews emphasize privacy-by-design architectures (e.g., edge processing) as a prerequisite when IAQ data are used to infer occupant behavior [14].

Despite this progress, pollutant-based activity recognition remains challenging in realistic deployments because indoor air quality time series are typically dominated by baseline periods. In real-life indoor environments, emission-generating events are sparse and episodic, while much of the recorded signal reflects background variability driven by ventilation, infiltration, occupancy and residual pollutant decay. Recent work in air-quality sensing has also highlighted that activity patterns and indoor source contributions must be explicitly considered to avoid exposure misclassification and to correctly interpret fixed-location sensor measurements [15]. As a result, a flat multi-class classifier that attempts to directly assign every temporal window to a specific activity may be forced to learn two distinct tasks at once: firstly, whether a window is informative or simply reflects baseline conditions, and secondly, which specific activity is responsible when a relevant event is present.

This formulation can reduce robustness in baseline-dominated environments. When most windows correspond to non-event conditions, ordinary background variability may be incorrectly mapped to activity labels, increasing false positives and destabilizing predictions. Moreover, indoor activity datasets are typically characterized by strong class imbalance and overlapping temporal signatures across activities, meaning that many windows are only weakly informative with respect to the target label. In such settings, reliable operation depends not only on discriminating among activity classes, but on explicitly separating activity-relevant dynamics from ordinary background behavior before attempting fine-grained classification.

In this work, we address this problem by reformulating pollutant-based activity recognition as a hierarchical gated inference task. Instead of directly applying a flat multi-class model, the proposed framework first determines whether a temporal window contains activity-relevant pollutant dynamics and only then performs fine-grained activity classification. The first stage acts as an activity-relevance gate that filters baseline windows, while the second stage conditionally identifies the specific activity class for windows that pass the gate. This decomposition better reflects the physical structure of indoor pollutant generation, where source events are intermittent and embedded within longer non-event periods.

The motivation for this approach is not only methodological but also application-driven. The World Health Organization continues to identify household air pollution as a major health concern and recent evidence shows that everyday indoor sources such as cooking and cleaning can substantially influence personal exposure to harmful pollutants [16]. A robust, privacy-preserving method for identifying these pollution-generating activities can therefore support exposure-aware monitoring, intelligent ventilation control, adaptive building operation, and health-oriented indoor environmental management.

Accordingly, this paper proposes a gated hierarchical framework for unobtrusive human activity recognition using multivariate IAQ sensing. Using time-windowed measurements of particulate matter, VOCs, CO_2_, temperature and relative humidity, the framework separates baseline detection from activity identification in order to improve robustness under realistic baseline-dominated conditions. By aligning the inference process with the temporal structure of indoor pollutant emissions, the proposed approach aims to reduce false activations while preserving reliable discrimination among pollutant-generating household activities.

The relevance of detecting indoor human activities from air quality signals lies in the fact that many everyday household actions are not only behavioral events but also pollutant-generating episodes with direct implications for exposure and environmental control. Activities such as cooking and cleaning can substantially alter concentrations of particulate matter, VOCs and combustion-related pollutants in the breathing zone, making their identification valuable for exposure-aware monitoring, adaptive ventilation and intelligent indoor environmental management. In this context, the goal is not activity recognition as an isolated pattern-recognition task, but the extraction of actionable information from pollutant dynamics using a sensing modality. 

Compared with previous pollutant-based activity recognition studies, the main contribution of this work is not only the use of multivariate IAQ sensing but the reformulation of the problem as a hierarchical inference task. Existing approaches commonly treat activity recognition as a flat multi-class classification problem, even though real indoor monitoring is dominated by long baseline periods. The proposed two-stage framework explicitly separates activity-relevance detection from activity identification, thereby aligning the recognition process with the physical structure of pollutant-generating events. This leads to a more robust treatment of baseline-dominated data and improves end-to-end activity recognition performance.

## 2. Materials and Methods

### 2.1. Portable Air Quality Monitoring System

The experimental data used in this study were collected using the portable air quality monitoring unit of the TwinAIR system, an integrated sensing framework developed to investigate the relationship between indoor air quality (IAQ), human activity and exposure. The portable device is designed for continuous real-world operation in residential and occupational indoor environments, while remaining unobtrusive to the user. The device is typically positioned close to the user’s breathing zone (e.g., mounted on the arm, worn as a pendant or placed nearby), allowing measurements representative of personal exposure rather than room-averaged air quality.

#### 2.1.1. Sensing Hardware and Measurement Principles

The portable unit continuously measures a set of environmental and pollutant variables selected to capture both emission processes and dispersion dynamics associated with indoor human activities. The monitored parameters include air temperature, relative humidity, carbon dioxide (CO_2_), volatile organic compounds (VOCs) and particulate matter (PM_1_, PM_2.5_, PM_4_, PM_10_).

Environmental conditions are measured using the SHT41 sensor (Sensirion AG, Stäfa, Switzerland), which provides high-accuracy temperature and humidity measurements through a CMOSens^®^ integrated sensing element protected by a PTFE membrane, allowing stable operation in particle-rich environments [17]. Gas-phase pollutants are measured using the SGP41 multi-gas metal-oxide (MOx) sensor (Sensirion AG, Stäfa, Switzerland). The device detects VOCs and NO_x_ through changes in the electrical resistance of a heated sensing layer caused by adsorption of reactive gases. The sensor output is processed using Gas Index Algorithm (Sensirion AG, Stäfa, Switzerland) to estimate VOC and NO_2_ indices suitable for continuous monitoring and detection of indoor emission events [18,19].

Carbon dioxide concentration is measured with the SCD40 sensor (Sensirion AG, Stäfa, Switzerland), based on a photoacoustic non-dispersive infrared (NDIR) principle. The sensor uses modulated infrared light absorption to quantify CO_2_ concentration and incorporates on-chip temperature and humidity compensation, providing stable measurements in dynamically changing indoor environments [20].

Particulate matter concentrations are measured using the SPS30 optical particle sensor (Sensirion AG, Stäfa, Switzerland), which operates on laser light-scattering. Suspended particles passing through the sensing chamber scatter laser radiation and the detected scattering intensity is used to estimate mass concentrations across multiple particle size fractions (PM_1_, PM_2.5_, PM_4_, PM_10_). The sensor provides calibrated measurements throughout its operational lifetime and is suitable for long-term monitoring applications [21].

The sensing subsystem was designed to ensure stable and temporally consistent acquisition of the variables used for activity inference. Sensor selection was based on digital sensors with manufacturer-calibrated outputs or manufacturer-supported signal-conditioning pipelines, allowing direct and repeatable acquisition of synchronized IAQ channels. Temperature and humidity were acquired from the SHT41, CO_2_ from the SCD40 with built-in environmental compensation, particulate matter from the SPS30 using calibrated digital PM outputs and gas-related variation from the SGP41 using the manufacturer’s Gas Index Algorithm to derive VOC and NOx signals. In the context of the present study, emphasis was placed on acquisition consistency, temporal alignment and repeatability across trials, since the downstream objective was the recognition of activity-related pollutant patterns rather than regulatory-grade estimation of absolute pollutant concentrations. Under this scope, the relevant requirement is that the sensing channels provide stable and comparable temporal responses to indoor events, which is distinct from the metrological validation procedures required for compliance-oriented reporting.

#### 2.1.2. Embedded Electronics and Communication

The sensing modules are interfaced to a central mainboard built around the STM32WL55 system-on-chip (STMicroelectronics, Geneva, Switzerland), which integrates a microcontroller and a sub-GHz LoRa radio transceiver [22]. The microcontroller manages the acquisition pipeline, including sensor polling, timestamp alignment, preprocessing, packet formation and communication. The sensing units are connected through digital interfaces, I^2^C and SPI, and are read within a common acquisition cycle so that all pollutant and environmental variables correspond to the same temporal context. This coordinated acquisition strategy ensures synchronization across sensing channels and measurement consistency in the resulting multivariate time series.

In the present study, the sensing cadence was set to 5 s, and this interval defines the temporal resolution of the data used to construct the sliding windows for model training and inference. However, this acquisition interval should not be conflated with the wireless communication schedule. The preprocessing required for window construction can be performed directly on the embedded platform, and therefore, a real-life implementation of the proposed technique would not require transmission of every individual 5 s sample. Instead, measurements could be buffered locally, assembled into windows on-device and transmitted in batches, with only the relevant context window sent to the backend. Under this deployment perspective, the 5 s interval is a sensing requirement for consistent temporal representation of indoor air-quality dynamics, rather than a strict requirement for LoRaWAN uplink periodicity.

The device is powered by a 1600 mAh rechargeable lithium-polymer battery and can also be charged through a USB Type-C connection or an integrated photovoltaic panel, supporting extended field deployments and long-duration monitoring campaigns. The selected sensing modalities collectively capture both emission and accumulation phenomena. Combustion and cooking activities primarily affect PM and NO_x_ levels, cleaning and solvent use affect VOC concentrations, and human occupancy and ventilation influence CO_2_ and humidity levels. By combining particulate, gaseous, and environmental measurements within the breathing zone, the device provides a multivariate signal describing indoor activity-related pollutant dynamics rather than relying on single-pollutant thresholds.

The selected sensing modalities collectively capture both emission and accumulation phenomena. Combustion and cooking activities primarily affect PM and NO_x_ levels, cleaning and solvent use affect VOC concentrations, and human occupancy and ventilation influence CO_2_ and humidity levels. By combining particulate, gaseous and environmental measurements within the breathing zone, the device provides a multivariate signal describing indoor activity-related pollutant dynamics rather than single-pollutant thresholds.

#### 2.1.3. Study Design and Experimental Protocol

The experimental protocol was designed to systematically observe indoor pollutant concentration dynamics before, during and after common household activities, under controlled yet realistic ventilation conditions. The primary objective was to capture reproducible pollutant “footprints” associated with specific activities, while also characterizing baseline behavior and post-activity decay under typical indoor operation.

Five household activities were selected and grouped into two categories based on their nature and expected emission mechanisms: cooking-related activities and cleaning-related activities. The cooking activities included boiling 1 L of water, pan frying protein and oven cooking of protein. The cleaning activities included vacuum cleaning and spray-based surface cleaning. These activities were chosen because they represent frequent indoor actions with documented impacts on indoor air pollutant concentrations.

Ventilation strategies were selected to reflect realistic usage patterns. During cooking activities, ventilation was provided by a range hood operating only for the duration of the activity. For cleaning activities, ventilation was achieved through an open window, which remained open for the entire duration of the experiment. No additional mechanical ventilation or air purification systems were used. Each activity–ventilation combination was repeated five times under identical conditions, with care taken to minimize external interference and background disturbances.

Cleaning activities were conducted in the dining room, while cooking activities were performed in the kitchen. These locations were selected to reflect typical spatial arrangements in residential environments and to ensure consistent geometry across repeated trials. The indoor air pollutants monitored during the experiments included particulate matter (PM_1_, PM_2.5_, PM_4_, PM_10_), VOCs, NO_x_, and CO_2_. Temperature and relative humidity were recorded concurrently to provide contextual information on indoor environmental conditions that may influence pollutant behavior.

#### 2.1.4. Sensor Placement and Data Collection

To approximate pollutant concentrations representative of personal exposure during household activities, the TwinAIR portable device was worn by the participant performing each experimental trial and positioned in the breathing-zone vicinity. Two realistic wearing configurations were considered during the campaign, namely pendant-style placement and arm-mounted placement using an armband. Both configurations were retained in the dataset in order to reflect practical wearable-use conditions rather than an artificially fixed laboratory arrangement. In all cases, the device remained in close proximity to the upper body throughout the monitored session, enabling continuous acquisition of pollutant concentrations and environmental conditions in the participant’s near field. The selected placement was informed by prior exposure-oriented analysis of pollutant transport and near-body airflow behavior (Computational Fluid Dynamics–CFD simulation), with the aim of capturing measurements more representative of personal exposure in the breathing-zone vicinity than of bulk room conditions. Each experimental recording was organized into three consecutive phases: a pre-activity baseline phase, an activity phase, and a post-activity observation phase. The sensing unit remained worn continuously throughout the full trial. Data collection began 20 min prior to activity onset, and this initial segment served as the explicit baseline reference period for characterizing background pollutant levels and short-term temporal variability in the absence of a deliberate pollutant-generating action. The 20 min duration was selected empirically, as repeated inspection of the recorded trials indicated that this interval was sufficient to capture stable baseline behavior prior to activity onset. In the present annotation scheme, this pre-activity segment constituted the baseline class, whereas post-activity windows were retained within the originating activity class in order to represent the full pollutant episode, including its decay.

Activity durations varied according to the nature of the task and were selected to reflect realistic execution times in everyday residential settings. Boiling, frying, and spray cleaning were typically completed within approximately 10 min, vacuum cleaning within approximately 15 min, and oven cooking within approximately 35 min. These durations were not imposed as rigid fixed intervals but reflected the normal execution of each task under the predefined experimental setup. Activity start and end times were annotated according to the experimental protocol timeline and were used to derive the temporal labels assigned to the acquired data.

Following the completion of each activity, monitoring continued for an additional 20 min in order to capture pollutant persistence, attenuation, and recovery under the corresponding indoor ventilation conditions. This post-activity period was included deliberately because, from an indoor air-quality and exposure perspective, the relevant episode does not end abruptly when the source-generating action stops; rather, pollutant concentrations remain elevated and continue to evolve through transport, ventilation, and removal processes that are causally linked to the preceding activity. The duration of the post-activity observation phase was defined empirically based on repeated examination of the recorded trials, which showed that the monitored signals returned toward background conditions within a broadly comparable temporal range after activity cessation. On this basis, a common post-activity interval was adopted as a consistent annotation rule across activities, allowing the decay phase to be represented in a uniform and reproducible way in the final dataset.

The resulting recordings therefore captured the full temporal evolution of each pollutant episode, including baseline conditions, activity-induced perturbation, and recovery dynamics. This design was considered particularly important for the intended recognition task, since both the onset and the attenuation of pollutant signatures may carry discriminative information relevant to activity inference. Under this wearable sensing configuration, the recorded particulate matter signals may reflect not only direct emissions associated with the household activity itself, but also motion-related effects such as particle resuspension and near-body airflow disturbance. In the present study, these effects were not treated purely as measurement artifacts, since the objective was to capture the pollutant dynamics experienced by the occupant under realistic use conditions. Accordingly, the PM signals should be interpreted as representing the combined outcome of source generation, indoor transport, and occupant-related disturbance within the personal exposure zone.

#### 2.1.5. Data Preparation for Activity Inference

The collected time-series data were preprocessed and segmented into consecutive temporal windows for subsequent analysis and modeling. Prior to segmentation, the data were chronologically sorted by timestamp, non-numeric values were coerced to numeric format, and missing values were imputed using the median of each feature, followed by zero filling where required. In addition, hour_of_day was introduced as an additional temporal context feature.

The multivariate IAQ time series was segmented into fixed-length temporal windows of 10 consecutive samples (L=10). This window length was selected as a compromise between capturing short-term pollutant dynamics, such as emission onsets, concentration ramps, and early decay patterns, and maintaining sufficient temporal resolution for near-real-time inference. Shorter windows were found to be more sensitive to noise, whereas longer windows reduced responsiveness and increased label ambiguity near activity boundaries. Windows were generated using a sliding-window approach with a stride of 1 s, resulting in strongly overlapping windows. This configuration was chosen to preserve temporal continuity, improve the representation of gradual pollutant variations, and support continuous prediction during real-time operation. Each window preserves the full temporal ordering of samples and the multivariate relationships among pollutant and environmental variables.

The final model input was represented as a three-dimensional tensor of shape N×10×10, where N denotes the number of windows, 10 the window length, and 10 the number of retained input features. The selected channels comprised Temperature, Relative Humidity, TVOC, ${CO}_{2}$, mass concentration of ${PM}_{1}$, mass concentration of ${PM}_{2.5}$, mass concentration of ${PM}_{4}$, mass concentration of ${PM}_{10}$, Particle Size, and hour_of_day.

To assign a single label to each window, labels corresponding to activity phases and specific activity classes were derived from the experimental timeline. This labeling process enabled supervised learning and evaluation of the proposed hierarchical inference framework, while ensuring consistency between the temporal evolution of pollutant signals and the annotated household activities.

This experimental setup and the resulting dataset build upon prior TwinAIR studies and provide a structured yet realistic basis for investigating pollutant-driven activity recognition under baseline-dominated indoor conditions.

### 2.2. Hierarchical Activity Recognition Architecture

#### 2.2.1. Problem Formulation

Let $X=\{x_{t}{\}}_{t=1}^{T}$ denote a multivariate indoor air quality time series, where each sample $x_{t}\in R^{d}$ comprises synchronized measurements of gaseous pollutants, particulate matter size fractions, and environmental variables. The objective is to infer, over time, whether an indoor activity is occurring and, if so, to identify the specific activity type responsible for the observed pollutant dynamics.

Rather than formulating this task as a flat multi-class classification problem over all temporal windows, we decompose it into two sequential inference stages:

1. Activity relevance detection (gate): determine whether a given temporal window corresponds to baseline conditions or contains activity-induced pollutant perturbations.
2. Activity identification (expert): conditionally classify the type of household activity, given that the window has been deemed activity-relevant.

This decomposition reflects the underlying generative process of indoor pollutant signals, in which emission events associated with human activities occur sparsely and are embedded within long baseline periods governed by ventilation, decay, and background variability.

#### 2.2.2. Temporal Windowing and Input Representation

The continuous IAQ time series was segmented into fixed-length overlapping temporal windows of length L, each comprising consecutive multivariate samples. Each window thus forms a three-dimensional tensor $W\in R^{L\times d}$, preserving the temporal ordering and cross-variable relationships of the pollutant signals.

Windowing serves two purposes. First, it enables the capture of short-term temporal dynamics, such as pollutant ramps, peaks, and decay patterns, which are characteristic of specific activities. Second, it provides a uniform input representation compatible with recurrent neural network architectures. Overlapping windows ensure temporal continuity and reduce sensitivity to window boundary effects.

#### 2.2.3. Stage 1: Activity Relevance Gate

The first stage of the architecture consists of a binary classification model that acts as a gate, assigning each temporal window to either a *baseline* or *activity-relevant* state. Baseline windows correspond to periods without deliberate emission-generating activities, while activity-relevant windows encompass time intervals during which pollutant dynamics are influenced by cooking or cleaning actions.

The gate is implemented as a recurrent neural network based on long short-term memory (LSTM) layers, selected for their ability to model temporal dependencies and non-linear dynamics in multivariate time series. Given an input window W, the gate outputs a probability score $p_{\mathrm{active}}\in [0,1]$, representing the likelihood that the window contains activity-related information. A decision threshold is applied to produce a binary gate output.

This stage is trained using labeled windows derived from the experimental timeline, with baseline labels assigned to pre-activity periods and activity-relevant labels assigned to windows overlapping the execution of household activities. Majority voting within each window is used to determine the final label, ensuring robustness to short transitional segments.

The primary role of the gate is not fine-grained discrimination, but rather reliable separation of informative and non-informative segments. By filtering out baseline windows early, the gate reduces class imbalance and prevents downstream classifiers from being exposed to overwhelming background variability.

#### 2.2.4. Stage 2: Conditional Activity Classifier

The second stage is a multi-class activity classifier that is invoked only for windows that pass the gate. Its task is to discriminate among the set of considered household activities based on their pollutant signatures. Activity classes correspond to the cooking and cleaning actions described in Section 3, excluding baseline conditions.

This classifier also employs an LSTM-based architecture, allowing it to learn activity-specific temporal patterns such as emission onset rates, peak structures, and decay behavior across pollutant channels. By conditioning its operation on the gate output, the classifier is trained and evaluated exclusively on activity-relevant windows, focusing its capacity on inter-activity discrimination rather than baseline rejection.

To ensure semantic consistency across activity phases, post-activity windows are mapped to the corresponding activity class, while pre-activity baseline windows are excluded from this stage. This normalization reflects the fact that pollutant decay following an activity remains causally linked to the originating action and can carry discriminative information.

#### 2.2.5. End-to-End Inference Procedure

During inference, the system processes the IAQ time series sequentially using the same windowing strategy as during training. For each window, the stage-1 gate first estimates the probability of activity relevance. Windows classified as baseline are assigned a null activity label, and no further processing is performed. For windows classified as activity-relevant, the stage-2 classifier predicts the most likely activity class.

The final output is a time-aligned sequence of labels indicating either baseline conditions or a specific household activity. This hierarchical inference strategy enables explicit control over false activations and missed detections and allows the system to be tuned according to application-specific priorities, such as sensitivity to short activities or robustness against background fluctuations.

#### 2.2.6. Rationale for Hierarchical Design

The proposed architecture introduces a structural prior that aligns the learning task with the physical characteristics of indoor pollutant generation and dispersion. By separating activity detection from activity identification, the system avoids forcing a single model to learn mutually incompatible decision boundaries under extreme class imbalance. Instead, each stage addresses a well-defined subproblem with clearer semantics and reduced complexity.

From a system perspective, the gated design also supports extensibility and deployment. Thresholds and temporal smoothing strategies can be applied at the gate level without retraining the activity classifier, and additional activity classes can be incorporated into the second stage with minimal impact on baseline discrimination. This makes the architecture particularly suitable for long-term indoor monitoring scenarios, where baseline conditions dominate and activity occurrences are sparse and episodic.

### 2.3. Model Training and Hyperparameter Configuration

#### 2.3.1. Temporal Segmentation and Window Construction

The multivariate IAQ time series was segmented into fixed-length temporal windows of 10 consecutive samples (L=10). This window length was selected as a compromise between capturing short-term pollutant dynamics, such as emission onsets, concentration ramps, and early decay patterns, and maintaining sufficient temporal resolution for near-real-time inference. Shorter windows were found to be more sensitive to noise, whereas longer windows reduced responsiveness and increased label ambiguity near activity boundaries.

Windows were generated using a sliding-window approach with a stride of 20 s, resulting in partially overlapping windows. This configuration was deliberately selected to reflect the intended continuous-monitoring deployment scenario, in which activity inference would be performed repeatedly as new measurements become available and predictions would therefore rely on temporally adjacent contexts rather than on fully disjoint segments. The use of overlap preserves temporal continuity, improves the representation of gradual pollutant variations, and increases robustness to arbitrary window boundaries. This is particularly important for short-lived or weakly expressed activity-related pollutant patterns, especially near activity onset and offset, where the informative portion of the signal may occupy only part of a window and could otherwise be underrepresented or missed entirely. Given the 5 s sensing cadence and the 10-sample window length, each window spans approximately 50 s of signal history; under a 20 s stride, consecutive windows overlap only partially, providing continuity without the excessively dense redundancy implied by near-sample-level shifting. Each window preserves the full temporal ordering of samples and the multivariate relationships among pollutant channels, yielding an input tensor of shape 10 × d, where d denotes the number of retained pollutant and environmental features.

To assign a single label to each window, majority voting was applied over the sample-level labels contained within that window. This strategy reduces labeling instability near activity transitions, where pollutant responses may lag behind the actual onset or cessation of an activity.

#### 2.3.2. Dataset Partitioning and Validation Strategy

The resulting windowed dataset was partitioned into 80% training data and 20% test data using a trial-based splitting strategy, with a fixed random seed to ensure reproducibility. In this context, a trial corresponds to a complete execution of a specific household activity under controlled experimental conditions. Each activity was repeated multiple times during the experimental campaign, and the sliding-window segmentation generated a large number of overlapping windows from each trial. Instead of randomly splitting individual windows, which could place highly similar or overlapping windows from the same activity execution in both training and test sets, the partitioning was performed at the trial level. Specifically, all windows originating from a given trial were assigned exclusively to either the training or the test subset. This ensures that temporally adjacent or strongly correlated windows derived from the same activity instance do not appear in both partitions, preventing potential information leakage caused by the overlapping-window segmentation. Importantly, this strategy does not exclude any activity class from the training process; each activity category is represented in both the training and test sets, but the test windows originate from different executions of the same activities compared to those used for training. This design provides a more reliable evaluation of the model’s ability to generalize unseen realizations of household activities while preserving the advantages of dense temporal sampling.

Within the training subset, 20% of the windows were further reserved for validation during training. This validation set was used exclusively for monitoring generalization performance and for triggering early stopping. No test data were used during model selection or hyperparameter tuning.

All reported performance metrics correspond to predictions on the held-out test set.

#### 2.3.3. Stage 1: Characteristics of the Activity Relevance Gate Model

The purpose of the activity relevance gate is to distinguish baseline conditions from activity-induced pollutant dynamics, independent of the specific activity type. This stage addresses the dominant class imbalance inherent in continuous indoor monitoring, where baseline periods occupy most of the time.

Windows were labeled as baseline or activity-relevant based on the experimental protocol. Windows overlapping with the execution of any household activity were labeled as activity-relevant, while windows drawn from the pre-activity baseline period were labeled as baseline. Transitional windows were labeled via majority voting to reduce temporal ambiguity.

Model Architecture

The gate model was implemented as a stacked LSTM network with the following configuration:

- Input layer: 10 × *d*;
- First LSTM layer: 150 units, return sequences enabled;
- Dropout: 0.4;
- Second LSTM layer: 150 units;
- Dropout: 0.4;
- Output layer: 2 neurons, softmax activation.

The use of stacked LSTM layers allows the model to learn both short-term fluctuations and higher-level temporal abstractions within each window. Dropout was applied between layers to reduce overfitting, given the strong temporal correlations induced by overlapping windows.

Training Configuration

The gate model was trained as a binary classifier using:

- Loss function: Sparse categorical cross-entropy;
- Optimizer: Adam;
- Batch size: 32;
- Maximum epochs: 100;
- Early stopping patience: 2 epochs, monitored on validation loss.

Early stopping was employed to prevent overfitting while allowing sufficient epochs for convergence. The model weights corresponding to the lowest validation loss were retained for inference.

During inference, the gate outputs a probability distribution over the two classes. The predicted class was obtained via argmax over the softmax output.

#### 2.3.4. Stage 2: Characteristics of the Conditional Activity Classifier Model

Training Set Construction

The activity classification model was trained exclusively on activity-relevant windows, as determined by ground-truth labels. Baseline windows were explicitly excluded to prevent the classifier from learning spurious decision boundaries dominated by background variability.

Pre-activity (“before”) windows were removed, as they represent baseline conditions by definition. Post-activity (“after”) windows were mapped to the corresponding activity class, reflecting the fact that pollutant decay dynamics remain causally linked to the originating activity and often carry discriminative temporal structure.

After preprocessing, the final activity label set consisted of five classes: boiling, frying, oven cooking, spray cleaning, and vacuum cleaning.

Model Architecture

The conditional activity classifier employed the following architecture:

- Input layer: 10 × *d*;
- First LSTM layer: 150 units, return sequences enabled;
- Dropout: 0.4;
- Second LSTM layer: 150 units;
- Dropout: 0.4;
- Output layer: 5 neurons, softmax activation.

This architecture mirrors that of the gate model to maintain architectural consistency while adapting the output layer to the multi-class setting.

Training Configuration

The activity classifier was trained using:

- Loss function: Sparse categorical cross-entropy;
- Optimizer: Adam;
- Batch size: 32;
- Maximum epochs: 50;
- Validation split: 20%;
- Early stopping patience: 2 epochs.

The reduced maximum number of epochs reflects the more homogeneous and balanced nature of the activity-only dataset, which typically converged faster than the gate model.

#### 2.3.5. Hierarchical Inference Procedure

During inference, the two models were applied sequentially to each temporal window using the same windowing configuration as during training. For each window:

1. The stage-1 gate estimated the probability that the window was activity-relevant.
2. If classified as baseline, the window was assigned a null activity label and excluded from further processing.
3. If classified as activity-relevant, the window was forwarded to the stage-2 activity classifier.
4. The activity classifier produced a probability distribution over the five activity classes, and the final label was selected via argmax.

This strictly hierarchical procedure ensures that fine-grained activity classification is performed only when sufficient evidence of activity-induced perturbation exists, reducing false alarms and improving robustness under baseline-dominated conditions.

## 3. Results

### 3.1. Ground Truth Definition and Temporal Alignment

The held-out test set comprised 1680 temporally aligned windows, of which 611 were baseline and 1069 were activity-relevant. Activity windows were distributed across boiling (*n* = 166), frying (*n* = 179), oven cooking (*n* = 333), spray cleaning (*n* = 186), and vacuum cleaning (*n* = 205). 

Window-level labels were derived from the experimental timeline using majority voting inside each 10-sample window to reduce boundary ambiguity and account for lagged pollutant responses. Baseline windows corresponded to the pre-activity segment, while windows spanning the activity execution and the post-activity decay period were assigned to the originating activity class to preserve the causal linkage between emission events and subsequent attenuation dynamics.

### 3.2. Evaluation of the Activity Relevance Gate

**Figure 1:**
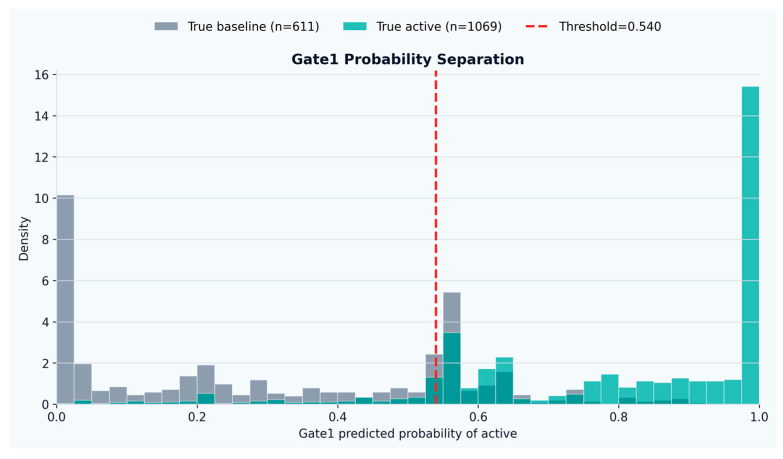
Probability separation of base gate.

Gate1 produced a strongly separated probability distribution between baseline and activity-relevant windows (presented in Figure 1), with baseline scores concentrated near 0 and activity scores concentrated near 1, and a limited overlap region around the selected operating threshold (*p*(active) ≥ 0.540).

**Figure 2:**
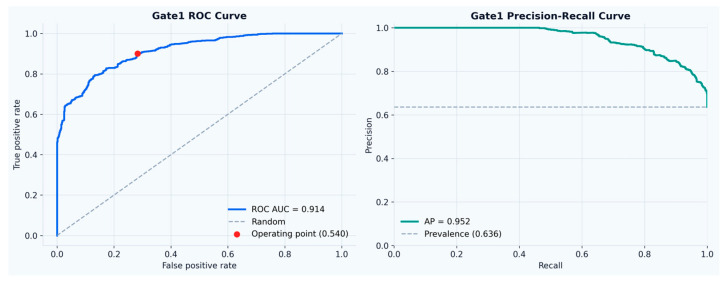
ROC and Precision-Recall curves.

**Figure 3:**
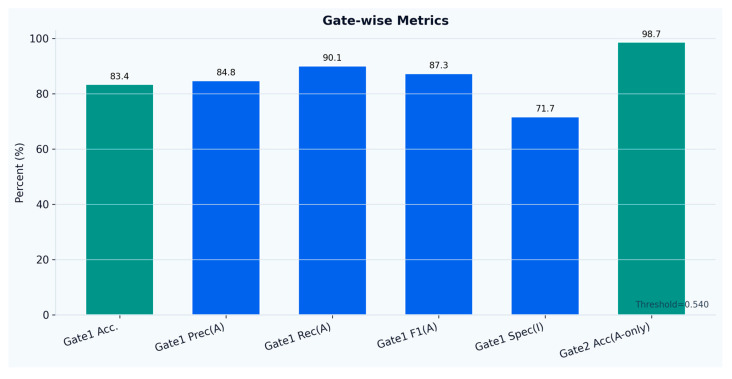
Evaluation metrics per gate.

Across the full test set, the gate achieved an ROC-AUC of 0.914 and an average precision (AP) of 0.952, substantially above the activity prevalence (0.636), indicating robust ranking performance for activity relevance, as presented in Figure 2. Additional metrics presented in Figure 3 are the first gate accuracy was 83.4%, with active precision 84.8%, active recall 90.1%, active F1-score 87.3% and baseline specificity 71.7%.

### 3.3. Evaluation of Activity Classification Performance (Conditional)

When evaluated exclusively on activity-relevant windows, the second-stage LSTM classifier achieved an accuracy of 98.7%. This suggests that, after the exclusion of baseline segments by the first-stage gate, the considered activity classes can be discriminated with very high reliability in the present dataset. Under this conditional evaluation setting, the remaining end-to-end errors of the hierarchical framework are mainly driven by the activity relevance gate, through false rejections of truly active windows and, to a lesser extent, false acceptances of baseline windows that are subsequently passed to the classifier.

### 3.4. End-to-End Hierarchical System Evaluation

**Figure 4:**
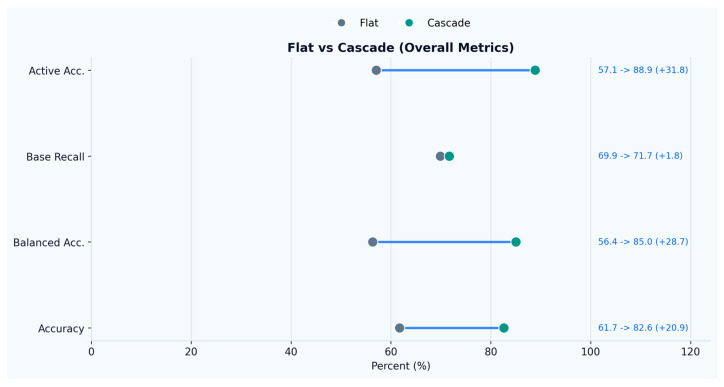
Evaluation metrics comparing the proposed approach to the baseline.

End-to-end evaluation treats a prediction as correct only when baseline windows are rejected (no activity emitted) and activity windows are both detected and assigned the correct activity label, with results presented in Figure 4. Under this strict criterion, the hierarchical cascade achieved 82.6% overall accuracy and 85.0% balanced accuracy (mean per-class recall across baseline and the five activities). For activity windows specifically, the end-to-end activity accuracy was 88.9%, while baseline recall remained 71.7%, indicating that improved activity recognition did not come at the expense of baseline rejection.

**Figure 5:**
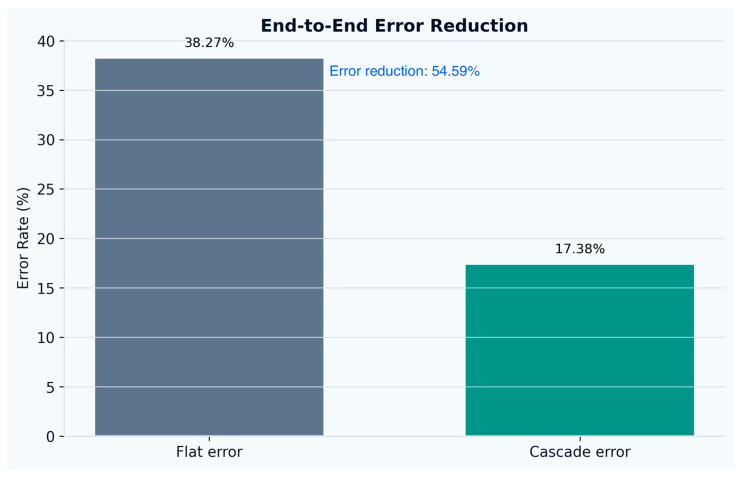
Overall end-to-end error reduction.

**Figure 6:**
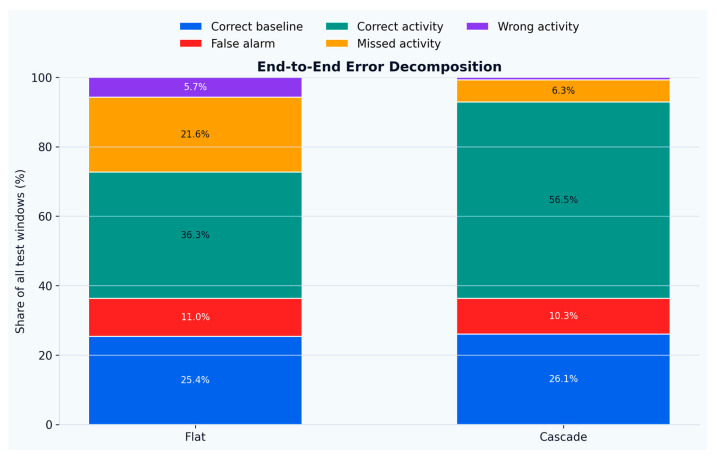
Error decomposition barplots.

Relative to the flat multi-class baseline, the hierarchical design reduced the overall error rate from 38.27% to 17.38% (54.59% relative error reduction), as shown in Figure 5. Error decomposition (Figure 6) shows that the main gain stemmed from a large reduction in missed activities (21.6% → 6.3% of all test windows) and wrong-activity assignments (5.7% → ~0.8%), while the false-alarm share remained comparable (11.0% → 10.3%). The share of correctly recognized activity windows increased from 36.3% to 56.5% of the full test stream, consistent with the improved base-vs-active trade-off achieved by the gated cascade.

### 3.5. Baseline Comparison: Flat Multi-Class Classification

**Figure 7:**
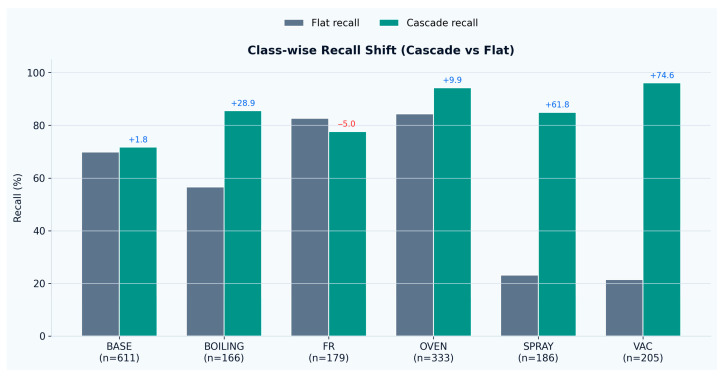
Class-wise Recall shift.

Using identical inputs and recurrent capacity, the flat classifier achieved 61.7% overall accuracy and 56.4% balanced accuracy. Its baseline recall was 69.9% (427/611 baseline windows correctly rejected), but activity accuracy was limited to 57.1% (610/1069 activity windows correctly labeled), reflecting the difficulty of jointly learning baseline rejection and fine-grained activity discrimination under baseline-dominated monitoring (Figure 7).

Class-wise analysis highlights that the cascade primarily improved the recognition of cleaning-related activities. Vacuum cleaning recall increased from 21.5% to 96.1% (+74.6 points), and spray cleaning recall increased from 23.1% to 84.9% (+61.8 points). Boiling recall also increased substantially (56.6% → 85.5%, +28.9 points), while oven cooking improved from 84.4% to 94.3% (+9.9 points) and baseline recall increased slightly (69.9% → 71.7%, +1.8 points). Frying recall decreased modestly (82.7% → 77.7%, −5.0 points), indicating that this class remains the most susceptible to confusion with baseline-like dynamics. This does not necessarily imply weaker frying-class discrimination in the hierarchical model itself; rather, unlike the flat classifier, the proposed two-stage approach requires frying windows to first be recognized as activity-relevant by the gate before activity labeling is performed. As a result, some borderline frying windows that may previously have been assigned directly to the frying class by the flat model were instead filtered out at the activity-relevance stage, reflecting a stricter separation between baseline and activity-relevant dynamics.

**Figure 8:**
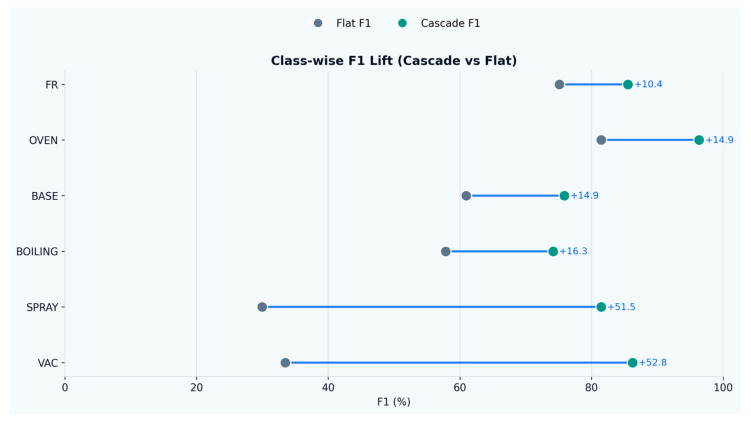
Class-wise F1 lift.

**Figure 9:**
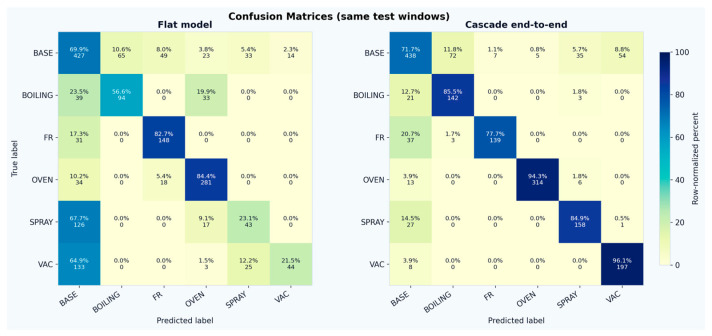
Confusion Matrices.

These shifts translate into large F1-score gains (Figure 8) for vacuum cleaning (+52.8 points) and spray cleaning (+51.5 points), with additional improvements for boiling (+16.3 points), baseline (+14.9 points), oven cooking (+14.9 points), and frying (+10.4 points). The confusion matrices show (Figure 9) that, for the flat model, most cleaning windows were absorbed into the baseline class (67.7% of spray and 64.9% of vacuum windows predicted as baseline), whereas the cascade greatly reduced this failure mode (spray-to-baseline 14.5% and vacuum-to-baseline 3.9%). Remaining errors in the cascade are concentrated in baseline–activity confusions near the gate threshold (e.g., 11.8% of baseline windows predicted as boiling and 8.8% as vacuum) and in frying windows that still map to baseline (20.7%), suggesting that future improvements should focus on separating low-emission cooking dynamics from background variability.

## 4. Discussion

The results support the central premise that indoor activity recognition from pollutant signals is structurally different from conventional flat multi-class HAR, because the dominant operating regime in real homes is “nothing happens” (baseline), disrupted by short emission events followed by decay. The hierarchical decomposition aligns the learning problem with that physical process. Stage 2 (activity identification) reaches near-ceiling performance when evaluated only on activity-relevant windows, which implies that the multivariate pollutant trajectories carry strong discriminative signatures for the considered activities once the model is not forced to simultaneously learn baseline rejection. In contrast, the flat formulation under the same recurrent capacity spends substantial representational efforts on separating baseline from “everything else,” and the confusion matrices show that it resolves ambiguity by collapsing many non-baseline windows into BASE.

The largest improvements occur for cleaning activities, where the flat model fails primarily by absorbing activity windows into baseline. Spray cleaning and vacuum cleaning are extreme cases: under the flat model, 67.7% of SPRAY and 64.9% of VAC windows are predicted as BASE, resulting in very low recall (23.1% and 21.5%, respectively). In the cascade, these failure modes are largely removed (SPRAY→BASE drops to 14.5%, VAC→BASE to 3.9%), and recall rises to 84.9% and 96.1%. This pattern is consistent with the hierarchical design goal: once the gate suppresses a large fraction of baseline variability, the second-stage classifier can focus on inter-activity structure and becomes much less prone to the “default-to-baseline” shortcut. Practically, this matters because cleaning-related exposures are often episodic, user-driven and potentially relevant to mitigation actions.

The gate is therefore the dominant contributor to end-to-end performance. Its operating point (threshold 0.540) yields high activity recall (90.1%) but only moderate baseline specificity (71.7%), which is reflected in the end-to-end confusion matrix, where baseline windows are sometimes forwarded and then assigned an activity label (notably BOILING and VAC). Because Stage 2 is highly accurate when invoked on truly active windows, tightening the threshold would primarily reduce false alarms at the expense of missing low-salience activity windows and on the contrary, relaxing it would increase sensitivity but propagate more baseline windows to the activity classifier, increasing false activity labeling. The reported error decomposition suggests that the chosen operating point strongly suppresses missed activities (21.6% → 6.3% of all windows) while leaving false alarms roughly comparable (11.0% → 10.3%), which is a reasonable deployment-oriented choice when the intent is to avoid missing emission events.

The choice to map post-activity decay windows to the originating activity class is defensible from both a causal and an application-oriented perspective. From an indoor air quality standpoint, the effect of an activity does not end abruptly when the source action stops, since pollutant concentrations remain elevated and continue to evolve through ventilation, dispersion, and removal processes that remain causally linked to the originating emission event. As such, decay dynamics may retain discriminative value, particularly for activities associated with different pollutant mixtures (e.g., VOC-heavy spray events versus PM-heavy vacuum resuspension), and may also remain relevant for exposure-aware monitoring and control-oriented applications. For this reason, post-activity windows were intentionally retained within the originating activity class rather than reassigned immediately to baseline. This annotation strategy is also consistent with an event-level interpretation of pollutant-generating activities, where the relevant phenomenon includes not only source activation but also the subsequent persistence of its impact on the indoor environment. Under this interpretation, the selected labeling approach provides a transparent and reproducible way to represent complete activity-induced IAQ episodes, especially in applications where the relevant output is the detection of a pollutant-generating event (e.g., ‘spray cleaning occurred around this time’) rather than a strictly instantaneous label assigned to each individual window.

From a systems perspective, the cascade has two additional advantages beyond accuracy. First, it enables explicit control over sensitivity via a single gate threshold without retraining the activity classifier, which is attractive for personalization (e.g., users with different ventilation habits). Second, it supports computational efficiency: Stage 2 is executed only on windows deemed active, which is aligned with battery-operated sensing and continuous back-end inference. Given the sampling cadence (5 s) and the 10-sample window length, the system can update predictions every sample once the window is filled, implying a practical temporal resolution on the order of seconds with a decision context on the order of ~50 s; this is compatible with near-real-time feedback while still capturing meaningful pollutant dynamics.

The main limitations are those expected for pollutant-driven recognition under controlled experiments. The activity set evaluated in the presented results includes five classes (boiling, frying, oven cooking, spray cleaning, vacuum cleaning) plus baseline. Generalization is also a key issue: the experiments are performed in a specific residential layout with fixed sensor placement rules, repeated trials and prescribed ventilation regimes (hood-only during cooking, window-open during cleaning). These constraints improve internal validity but may not reflect the diversity of real homes, products, and occupant behaviors. In addition, because the sensing unit is worn in the breathing-zone vicinity, particulate matter readings may also be influenced by motion-related effects such as particle resuspension and near-body airflow disturbances, especially during activities involving locomotion or mechanical agitation, such as vacuum cleaning. In the context of the present study, however, these effects were not treated purely as artifacts, since they form part of the realistic pollutant dynamics experienced by the occupant during the activity. Finally, the current evaluation is window-based; for real deployment claims, it is typically valuable to complement this with event-level metrics (false events per hour/day, onset detection latency, event fragmentation), since downstream applications generally act on detected events rather than isolated windows.

## 5. Conclusions

This work demonstrates that household activity recognition can be performed using multivariate indoor pollutant dynamics captured by a portable exposure-oriented sensing unit, and that a hierarchical inference structure is substantially more effective than a flat multi-class formulation under baseline-dominated monitoring. The proposed cascade separates “activity relevance” from “activity identification,” achieving strong gate ranking performance (ROC-AUC 0.914, AP 0.952) and near-ceiling conditional classification accuracy. End-to-end, the cascade improves overall accuracy to 82.6% and balanced accuracy to 85.0%, while reducing the end-to-end error rate by 54.6% relative to the flat model. The improvements are driven primarily by recovering cleaning activities that a flat model tends to collapse into baseline, raising vacuum and spray cleaning recall from ~21–23% to ~85–96% on the same test windows.

The remaining errors are concentrated in the baseline–activity boundary (false activations of baseline and missed low-salience segments), with frying being the most challenging activity under the selected operating point. Future work should extend the validated activity set, report event-level performance on continuous streams and evaluate generalization across different homes, ventilation patterns and product/recipe variability.

The results support the central premise that indoor activity recognition from pollutant signals is structurally different from conventional flat multi-class HAR, because the dominant operating regime in real homes is baseline behavior, interrupted by relatively short emission events and their subsequent decay. The proposed hierarchical decomposition aligns the learning task with this physical process by separating activity-relevance detection from activity identification. In this respect, the study demonstrates the algorithmic validity of the proposed approach, showing that pollutant-driven human activity recognition can be performed with high accuracy when supported by an appropriately annotated dataset. The practical relevance of the framework should therefore be understood primarily in the context of calibrated or personalized deployment rather than universal out-of-the-box use across arbitrary homes. In real-world settings, pollutant signatures are inherently shaped by home layout, ventilation practices, occupant behavior, and the specific products or appliances involved, making some form of environment-specific calibration a realistic requirement rather than a weakness unique to the present method. At the same time, the framework remains practically valuable even when fine-grained activity labeling is not the sole objective. In particular, the first-stage gate can already function as a reliable detector of pollutant-generating events, supporting applications such as adaptive ventilation or air-cleaning control, exposure-aware monitoring, and automatic segmentation of relevant IAQ episodes in continuous recordings. From this perspective, the main contribution of the work is not the claim of universal cross-home generalization without adaptation, but the demonstration of a robust recognition architecture that can serve as the inference core of home-specific unobtrusive monitoring systems. Nevertheless, some limitations should be acknowledged. The present evaluation considers a restricted activity set consisting of five household activities, namely boiling, frying, oven cooking, spray cleaning, and vacuum cleaning, in addition to baseline conditions. Furthermore, the experiments were conducted under controlled conditions in a specific residential layout, with fixed sensor placement, repeated trials, and predefined ventilation regimes, choices that strengthen internal validity but do not fully capture the diversity of real homes and occupant practices. Finally, the evaluation is performed at the window level; for deployment-oriented assessment, future work should complement these results with event-level metrics, such as false events per hour or day, onset detection latency, and event fragmentation, since downstream control and monitoring applications typically operate on detected events rather than isolated windows.

## Data Availability

The source code is available on request from the corresponding author.
